# Oversimplification and Overstandardization in Biological Methods: Sperm Bioassays in Ecotoxicology as a Case of Study and a Proposal for Their Reformulation

**DOI:** 10.1155/2014/936202

**Published:** 2014-02-06

**Authors:** M. A. Murado, M. A. Prieto

**Affiliations:** Grupo de Reciclado e Valorización de Materiais Residuais (REVAL), Instituto de Investigacións Mariñas (CSIC), r/Eduardo Cabello 6, Vigo, 36208 Galicia, Spain

## Abstract

An interesting toxicological bioassay (fertilization inhibition in sea urchin) uses as assessment criterion a variable (fertilization ratio) whose variation with time creates two types of difficulties. First, it fails to distinguish between the toxic effect and the spontaneous decline in the sperm activity, causing some inconsistencies. Second, the sensitivity of the fertilization ratio to many other variables of the system requires a complex standardization, constraining the achievement of the method without solving its main problem. Our proposal consists of using a parameter (sperm half-life) as the response of the assay, and describing explicitly the behavior of the system as a simultaneous function of dose and time. This new focus is able to solve the problematic character of the results based on the fertilization ratio and by using the same data set which is required by the conventional approach; it simplifies the protocol, economizes experimental effort, provides unambiguous and robust results, and contributes to the detection of an artefactual temperature effect, which is not very evident under the usual perspective. Potential application of this new approach to the improvement of other formally similar bioassays is finally suggested.

## 1. Introduction

In the study of biological systems, at times a defined gap exists between the recognition of the complexity, which should be accepted for describing certain phenomena in a reasonably realistic way, and the oversimplifications often applied to basic problems, frequently involving important practical consequences. Usually such gaps are not justified by differences between the levels of study, since a simpler approach from a factual point of view does not involve necessarily simpler formal tools. The more common reasons argued in this regard are related to the supposed usefulness of routines which can be solved in practically automatic ways to favour their reproducibility. However, this advantage disappears when—as it is very usual—the routine leads to the need of standardizing many variables or when—as it occurs occasionally as well—its original purpose is distorted.

Under this perspective, we will discuss and propose new focus for a useful bioassay which is based on the drop of the fertilizing success of free spawners in the presence of a toxic agent and is applied since years ago in the ecotoxicological field. This method, with interesting capabilities, was initially designed for sea urchin, in which the immediate formation of the fertilizing membrane around the egg makes the detection of the products of the process easy. Afterwards, the procedure has been applied to other organisms, such as the coral *Acropora millepora* [1] or the polychaete *Hydroides elegans* [2], and it is quite clear that the essence of the bioassay—and its problems as well—can be generalized to very different situations from the original one.

## 2. Theoretical Background and Methods

### 2.1. The Conventional Approach

The current procedure, with precedents since almost one century ago [3], is a synthesis of those that were developed by Dinnel et al. [4–6] and Pagano et al. [7–9], and it is recommended at present by environmental agencies [10]. The bioassay involves the exposure of a sperm suspension to increasing levels of a toxic agent, during increasing times, followed by the addition of an egg suspension to the treated sperm [11, 12]. After the time required to reach the asymptotic maximum of the fertilization ratio in the absence of the agent (control), the products of the process are fixed with formalin and counted. Subsequently, the toxic effect of the increasing doses of the agent over the fertilization ratio is assessed through any dose-response (DR) model.

Thus, if *F*_0_ and *F*_*c*_ are the fertilization ratios in the absence of toxic (control) and at the toxic concentration (dose) *c*, respectively, the response, quantified as *R* = 1 − (*F*_*c*_/*F*_0_), can be described with the Weibull dose-response model, as an example, which is an especially versatile DR model [13–16]. Using this equation in a reparametrized form [17–19], which makes it appropriate in this context, we can write

(1) $$\begin{array}{c} R=K\left[1-\exp\left(\ln0.5{\left(\frac{D}{m}\right)}^{a}\right)\right], \end{array}$$

where *K* is the asymptotic maximum of *R*, *D* the dose, *m* the dose corresponding to half-maximum response (*m* = ED_50_ if *K* = 1), and *a* a shape parameter that defines (together with *K* and *m*) the maximum slope of the function.

It should be underlined that this assay solves elegantly several key issues of the DR analysis: (a) it works with a large population, a condition that is difficult to satisfy if the target species is not a microorganism; (b) it deals with an ontogenically essential and physiologically sensitive process; (c) it is a fast test, avoiding the changes in the physical-chemical system [5] or in the biotic sensitivity [20] which are possible in longer assays, such as those that are focused on larval growth inhibitions. Short times are specially interesting in the assessment of lipophilic toxics, whose micelles can coalesce during the course of static assays lasting a number of hours, causing uncertainty regarding the real dose in the immediate environment of the organism.

However, the direct use of the drop in the fertilization ratio as response pays a steep price in practical complications and ambiguity of the results, both because of the characteristics of the system and the structure of its formal treatment.

#### 2.1.1. Exigencies of the Biologic System

Some necessary cautions in this regard are of a common kind in many analytical methods. This is the case, for example, of the effects of state variables as temperature and composition (essentially pH and salinity) or the need to use glass material [5]. A fact that has also correlates in other methods is the requirement, for maximizing the sensitivity, of a fertilization ratio for the control close to 1, but less than 1, to avoid as much as possible hiding the toxic effect by a possible excess of sperm. However, in a more specific and obstructive way, the assay is affected by particular variables, such as: (1) the absolute and relative gamete concentrations; (2) the contact time; (3) the sperm age; (4) the dilution of the system accelerates the consumption of its limited energy reserve, probably due to an increase of respiration, and therefore shorts out its life span. The control of these last factors is difficult, because the existence of interactions among them [21] prevents the individualized selection of appropriate values. In fact, standardization is a customary claim in the bibliography regarding this method [22–24], and other authors [25] even have argued the need to use more than one gamete ratio to take into account the reproductive failure due to polyspermy.

#### 2.1.2. Implications of the Descriptive Approach

Under this point of view, the method shows the following problematic aspects.

1. Although it is recognized that the exposure time to toxic agent affects the fertilization ratio [5], this variable is not formally included into univariate DR models as (1). Therefore, since each time leads to a different assessment, the decision about which is more representative is arbitrary.
2. The short life span of the gametes makes feasible a more realistic assay, in which the exposure time cover as their entire life period, as it occurs in natural conditions. The use of briefer exposure times can only contribute to increase the error and underestimate the toxic effect.
3. If the sum of the exposure and contact times does not exceed the sperm life span, it could happen (assuming that the toxic action reduces the sperm activity) that, at the end point, the fertilization processes are, under different doses, at different distances from their respective maxima, making any comparison questionable.
4. The values of the fertilization ratio *F* contain information regarding the effects of the toxic action and the sperm age, but the formal treatment ignores the second one. Thus, the variability linked to both effects is accumulated only over the toxic action, causing two undesirable consequences: the inaccuracy of the result and the bias of the parametric estimates.
5. Finally, as we will see in the results section, temperature exerts an inevitable and purely artefactual effect—that is apart from the one ruled by the Arrhenius equation—in any assessment through the conventional method.

### 2.2. Factual Frame of the Assay: Fertilization Kinetics

In the reproductive phenomenology of the sea urchin, rich in studies, the most of its quantitative behavior, both in experimental [21] and observational [26] contexts, is explained by a fertilization kinetic model which Vogel et al. [27] called—a curious tribute to Mozart—*Don Ottavio*. This model assumes that the random encounter between gametes follows a second order kinetics, in which the egg retains a certain number of spermatozoa, irrespective of the fertilizing character of the event. Thus, the fertilization ratio (*F*), *S*_0_ and *O*_0_ being the initial concentrations of gametes, can be described as

(2) $$\begin{array}{c} F\left(t\right)=1-\exp\left\{-\frac{\beta }{\beta_{0}}\frac{S_{0}}{O_{0}}\left[1-\exp\left(-\beta_{0}O_{0}t\right)\right]\right\}, \end{array}$$

where the kinetic constants *β* and *β*_0_ (mm^3^·s^−1^: required volume for a fertilizing rate of one egg per second) are the product of the sperm speed by the total (in *β*_0_) or effective (in *β*) egg section.

Several authors [25, 26, 28] have pointed out that this model does not take into account the fertility failures due to polyspermy induced by high *G*_0_ = *S*_0_/*O*_0_ ratios, which produce no asymptotic curves, but with a drop after a maximum. However, this problem is outside the strict kinetic process, and therefore (2) can provide a useful perspective for guiding the assay considered here.

**Figure 1:**
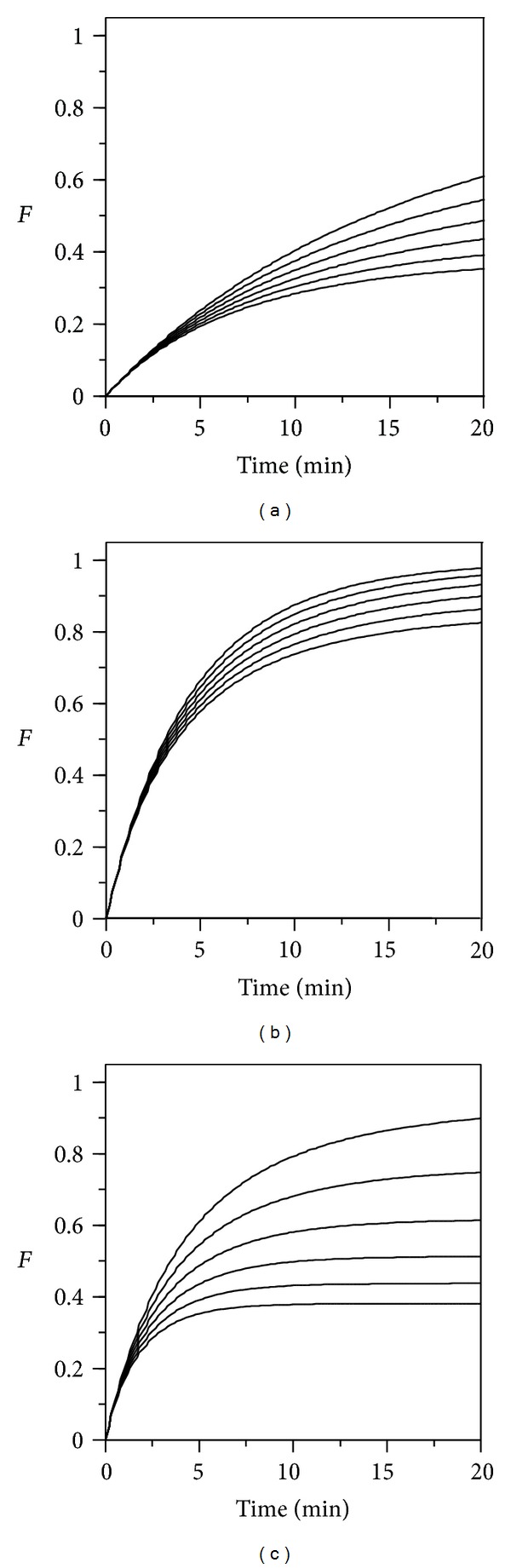
Effect of the parameters *β* and *β*_0_ from (2) on the fertilization kinetics for a constant value of *S*_0_ (1,000/*μ*L) and increasing values of *O*_0_ (1, 2,…,6/*μ*L). (b) Parametric values (*β* = 3.8 × 10^−6^; *β*_0_ = 3.3 × 10^−4^ mm^3^·s^−1^) from Vogel et al. [27]; at left and right, results of dividing by 4 the value of *β* and multiplying by 4 the value of *β*_0_, respectively.

If the idea is to base the evaluation on parametric variations, a first option (not very feasible, but that needs to be discussed) would be to use the parameters *β* and *β*_0_ of (2), both dependent on the sperm activity, which is the sensitive variable of the system. As illustrated in Figure 1, the fertilization rate and the asymptotic value of *F* decrease if *β* does it, and the asymptotic value decreases if  *β*_0_ increases. Thus, at a given *G*_0_ ratio, the kinetic data at different doses of a toxic agent would able to assess the toxic effect on both parameters.

This approach could be simplified by taking into account that at the high *G*_0_ ratios which are used in practice, the process can be considered as following a pseudo-first order kinetics. In fact, all profiles of Figure 1 could be adjusted with a high accuracy (*r* ^2^ > 0.999) to the model:

(3) $$\begin{array}{c} F=F_{\infty }\left[1-\exp\left(-\mu \cdot t\right)\right], \end{array}$$

where the parameters *F*_*∞*_ and *μ*, both potentially sensitive to the toxic effect, are the asymptotic maximum of *F* and the maximum specific rate of fertilization, respectively.

**Figure 2:**
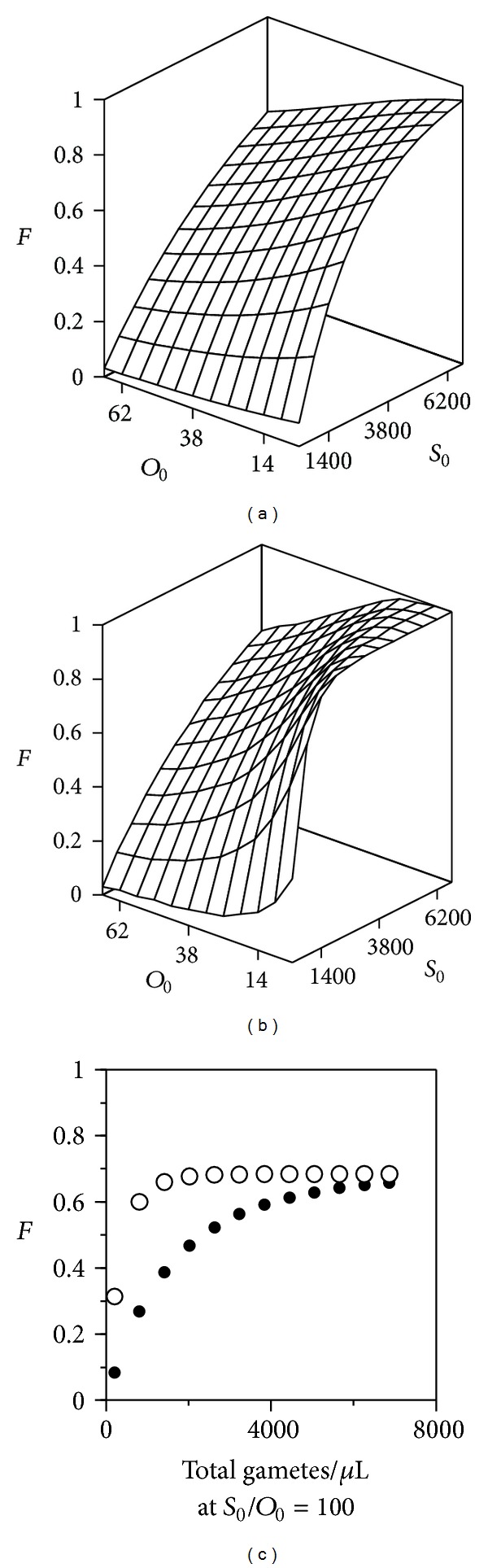
Fertilization ratio as a function of gamete concentrations in *Paracentrotus lividus* ((2) with the central parametric values from Figure 1) at 2 (a) and 10 minutes (b). (c) Values along the diagonal of the *S*_0_ *O*_0_ plane in (a) (∙) and (b) (∘), illustrating the effect of the absolute gamete population for a single *S*_0_/*O*_0_ ratio.

However, the use of either of these parametric pairs (both *β*, *β*_0_ and *F*_*∞*_, *μ*) has several disadvantages. One is the sensitivity of the required data, measured in a relatively short time interval, to the experimental error. Another one is the dependence of the parametric values on the *G*_0_ ratio (Figure 2, which also shows that the effect of the overall gamete population becomes less relevant as the contact time increases). Finally, the variations of each parametric pair are in general strongly correlated. Therefore, solving separately the kinetic and DR models would be a requirement, which would result in losing the advantages of a simultaneous solution as it will be proposed next, making use of another aspect of the work of Vogel et al. [27].

### 2.3. An Alternative Proposal

The main issue of the bioassay here studied is the fact that the sperm activity declines simultaneously with age and the toxic action, within the same timeframe and following in both cases a sigmoidal profile. Our proposal consists of accepting this dualism and submitting it to a model able to describe simultaneously, but in a distinctive way, both phenomena. It requires defining the response not as a function of the fertilization ratio (*F*), which varies with time, but as a function of a time parameter, such as the sperm half-life (*τ*).

The sperm life span—and therefore its half-life—is determined through the drop of the fertilization ratio with time, which Vogel et al. [27] described with the normal mass function. As this function lacks in the explicit algebraic form that is required for our purpose, we will use the Weibull mass function, that is, (1), now in its decreasing form:

(4) $$\begin{array}{c} F=F_{m}\exp\left(\ln0.5{\left(\frac{t}{\tau }\right)}^{v}\right), \end{array}$$

where *F*_*m*_ is the initial maximum of *F*, *t* is time, *τ* is the half-life, and *v* is the shape parameter. It should be noted that *F* varies between a maximum value *F*_*m*_ at age zero (independent of the toxic agent, because it implies a null exposure time) and a null value when the sperm exhausts its life span (at a dose-dependent time). Thus, it can be considered that *F*_*m*_ = 1, and then expression (4) is reduced to

(5) $$\begin{array}{c} F=\exp\left[\ln0.5{\left(\frac{t}{\tau }\right)}^{v}\right]. \end{array}$$

**Figure 3:**
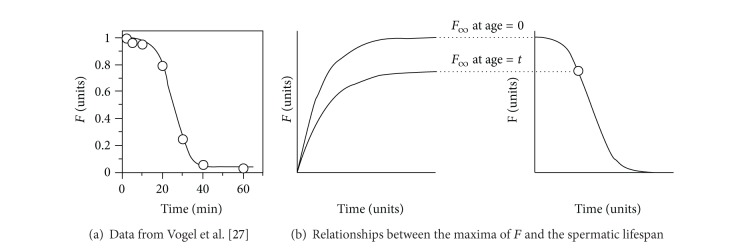
(a) Spermatic lifespan (*Paracentrotus lividus*) in sea water at 18–20°C, pH = 8.2-8.3, diluted “dry” sperm (1/3,000). Data from Vogel et al. [27] (points), adjusted to (5) (line). (b) Relationships between the maxima corresponding to the fertilization kinetics (left) and the spermatic lifespan (right).

When this equation was applied to the data from [27], the resulting *τ* value of 25.09 ± 1.45 minutes was in good agreement (Figure 3(a)) with the result obtained by these authors using the normal distribution (*τ* = 25.0 ± 8.2 minutes).

Now, if *τ* decreases from a *τ*_0_ value in the absence of toxic to a *τ* value in the presence of a given dose of the studied toxic, the response *R*_*τ*_ can be formulated as

(6) $$\begin{array}{c} R_{\tau }=1-\frac{\tau }{\tau_{0}},\;\text{therefore}\;\;\tau =\tau_{0}\left(1-R_{\tau }\right), \end{array}$$

where *R*_*τ*_ is (1). Thus, the bivariate model

(7) $$\begin{array}{c} F=\exp\left[\ln0.5{\left(\frac{t}{\tau }\right)}^{v}\right],\\ \tau =\tau_{0}\left\{1-K\left[1-\exp\left(\ln0.5{\left(\frac{D}{m}\right)}^{a}\right)\right]\right\} \end{array}$$

should provide an unambiguous evaluation of the toxic effect on the sperm half-life.

Since *τ* is determined from the maximum value of *F* at different sperm ages, from now on a distinction should be made between two maxima of *F* with different meaning (Figure 3(b)). *F*_*∞*_ is the asymptotic maximum of *F*, obtained after enough contact time (*t*_*∞*_) at any working conditions, for example, at different sperm ages. *F*_*m*_ is the initial maximum value corresponding to *F*_*∞*_ at the age which is considered as zero.

### 2.4. Experimental Procedure and Standardization Needs

The experimental protocol should observe the same cautions as the conventional one regarding the manipulation of the gamete suspensions, and it differs very little from this last one regarding its execution. In the beginning of the test, the following materials—volumes are only indicative—should be prepared: (i) a suspension of sperm and another one of eggs at the appropriate concentrations (see below) for the assay; (ii) *n* series of *k* tubes each: *k* − 1 doses of the toxic agent in 9.5 mL of seawater and one control. Under these conditions, the bioassay involves the following:

1. at time zero, to add 100 *μ*L of the sperm suspension to all tubes;
2. at increasing times (including zero), covering the entire sperm life span estimated for control, to initiate fertilization in the corresponding *n*_*i*_ series by adding 400 *μ*L of the egg suspension;
3. after a sufficient contact time to reach, in each *n*_*i*_ series, the asymptotic value *F*_*∞*_ in control to fix the products of the process by adding 100 *μ*L of formalin solution.

The use of the parameter *τ*, instead of the variable *F*, as the basis of the assessment offers here another important advantage. Indeed, *F* is a sensitive value to the initial gamete ratio (*G*_0_ = *S*_0_/*O*_0_) and, in fact, in assays based on the variation of *F*, Dinnel et al. [5] stated that the sensitivity to the toxic agent is inversely correlated with *G*_0_ ratio. However, if, within a wide range of *G*_0_ values (with constant *S*_0_), *F*_*∞*_ at age zero (i.e., *F*_*m*_) is coded as 1, in all cases the same value of *τ* is obtained, which makes this criterion very robust against variations of *G*_0_ ratio.

In fact, the only particular variable of the system that affects the sperm half-life is the sperm dilution, due to its role in the oxygen availability, as described by Levitan et al. [21]. Thus, the only condition that determines the appropriate *G*_0_ ratio is the need to avoid a sperm excess that could hide the toxic effect. This is achieved if *F*_*m*_ < 1, what does not prevent to code this value as 1. Dinnel et al. [5] used values in the reasonable range [0.6; 0.9], but it should be noted that the closer *F*_*m*_ is to 1, the clearer results are obtained.

**Figure 4:**
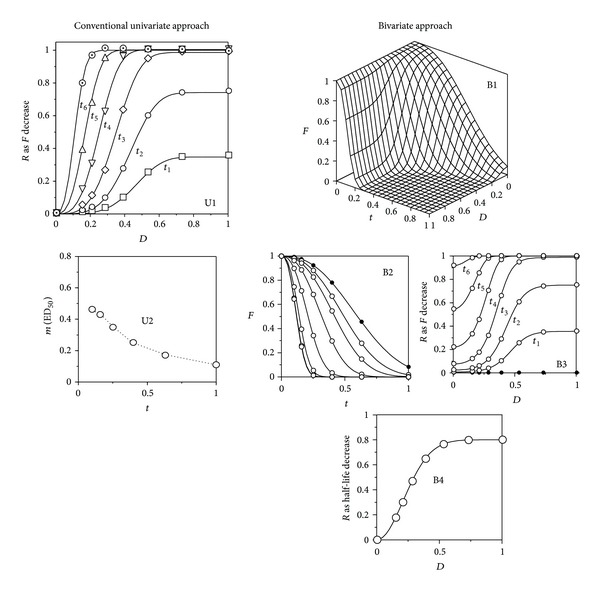
Different perspectives of the relationships among dose (*D*), exposure time (*t*), fertilization ratio (*F*), and response (*R*), this last one defined as (increasing) decrease of *τ* or *F*, as a function of the dose with respect to the control. Simulations from model (7) with the parametric values are specified in Table 1, supposing a negligible error (*σ* = 5 × 10^−4^). The closed symbols in subfigures (B2) and (B3) correspond to the control time course and the complete dose series at time zero, respectively. The open symbols in subfigures (B2) and (B3) correspond to the different response at different doses of toxic and the different exposure times, respectively. See text and Table 1 for details.

Clarity makes equally advisable low working temperatures, which extend the sperm half-life and provide a “space” (see Figure 4(B2)) for life spans that are shortened by the toxic action. Later on, another important implication of the temperature will be discussed.

The test is also consistent against objections about the possible effects of polyspermy [25, 26, 28], because the sperm half-life is not related to subsequent fertilization failures or larval malformations (relevant in larval assays) which can derive from a multifertilized egg.

### 2.5. Numerical Methods for Comparing the Two Approaches

A comparison between the described alternatives through a reasonable experimental effort would lack statistical reliability. Another solution is to use simulation experiments with realistic values, including error. As we shall see later, such a solution is especially appropriate in the present case.

Simulations were carried out by assigning concrete parametric values to model (7) to generate, in a *Microsoft Excel* spreadsheet, virtual assays with 8 doses at 7 times, including zero in both cases. An *ad hoc* macro was written to execute series of 2,000 virtual assays, each of them involving the following operations: (1) addition of a normal homoscedastic error to the model-generated values of *F*; (2) fitting of the result to models (1) and (7) to estimate their parameters by nonlinear least squares (quasi-Newton), through the *Solver* complement included into *Microsoft Excel*; (3) calculation of the parametric confidence intervals (Student's *t*-test, with *α* = 0.5) by applying *Solver Aid* macro [29].

To facilitate comparisons, doses, times, and fertilization ratios were coded into the interval [0; 1]. Since the response is sigmoidal both as a function of dose and time, the values of both variables were established according to a geometric progression with a ratio *g* = (1/*x*_1_)^1/(*n*−2)^, where *x*_1_ is the first non-null term of the series and *n* the number of terms, including zero. The experimental error was simulated with random normal numbers *N* : (0; *σ*) as described previously [30], by using the following expression:

(8) $$\begin{array}{c} N:\left(0;\sigma \right)=\sigma \left[{\left(-2\ln u_{1}\right)}^{1/2}\right]\sin\left(2\pi u_{2}\right), \end{array}$$

where *u*_1_ and *u*_2_ are two random uniform numbers as provided by the spreadsheet. The values routinely assigned to *σ* were 0.05-(0.025)-0.15 and, for some cases, *σ* = 0.25 was reached (i.e., from 5 to 25% of the maximum value of the dependent variable *F*). Also for clarity, we have used the notation CI for the confidence semi-interval as % of the parametric value. Thus, in the usual expression *θ* ± CI, *θ* estimate is statistically significant only when CI < 100.

Since fittings were carried out with *α* = 0.05 in the Student's *t*-test, a virtual series is considered statistically significant (95%) when 100% of the 2,000 repetitions are significant. Now, the parametric CI can be calculated according to two criteria: (1) averaging the CI resulting from the 2,000 repetitions; (2) calculating them (*α* = 0.05) on the basis of the 2,000 parametric estimates. Although both criteria differ very little, the second one is slightly more concessive, and it was not applied. Skewness and kurtosis coefficients of the parametric distributions were calculated using all estimates, significant or not. Although they are informative values, it should be kept in mind that their basis on the moments of third and fourth order tends to exaggerate the effect of the more deviant estimates from mean.

## 3. Results and Discussion

Conventional (M0) and alternative (M1) methods use the same data sets: fertilization ratios (*F*_*c*,*t*_) at a toxic concentration *c* and a time *t*. However, the different formal frames in which they are processed make the nature of the respective results different.

In M0, DR model (1) is individually applied, at each time, to a response defined as *R*_*F*_ = 1 − (*F*_*c*,*t*_/*F*_0,*t*_), that is, as the decrease—increasing with the dose—of the fertilization ratio at a given time. In M1, model (7) is directly applied to the whole of the *F*_*c*,*t*_ values, and the response, increasing with the dose as well, is the decrease of the sperm half-life. As far as here, we have used the same parametric notation (*K*, *m*, *a*) in DR model (1) and in the DR part (second equation) of model (7). From now on, if necessary, we will distinguish between both meanings by using the subscripts *F* and *τ*.

The fact that M0 and M1 use the same observational values within different conceptual frames has an important consequence for the validation (or refutation) of M1, since this approach is under the obligation to explain the results from M0, as well. If it is so, the abundant experimental results which have led to accept M0 become an experimental validation of M1. And in such a case, the selection of one or another approach is reduced to compare the logical consistence of their conclusions as well as the statistical reliability of results that are affected by the same error in the dependent variable (*F*_*c*,*t*_).

### 3.1. Relationships between the Conventional and Alternative Assays

Firstly, to clarify the ideal relationships between the two descriptions, both were applied to a set of simulations carried out by assigning the parametric values specified in Table 1 to model (7), supposing an assay without replicates with a negligible, but nonnull, error (*σ* = 5 × 10^−4^), to allow the running of the statistical tests.

Results (Table 1 and Figure 4) showed that the assessments derived from the M0 approach are exactly as those described in bibliography. Dinnel et al. [5], for example, working with silver nitrate as a toxic agent, specified that the “*fertilization success was inversely related to sperm exposure time*” and, indeed, the decrease with time of the values of *m*_*F*_(ED_50,*F*_) could be described with a hyperbolic equation (Figure 4(U2)). Such a description, however, is not very interesting, since the notion of *m*_*F*_ is meaningless both at zero time and at times beyond the sperm life span, preventing the existence of useful reference points.

Since a simulation with model (7) produces, through model (1), the typical results of the M0 approach, the descriptive capability above required for accepting M1 is proven. Thus, it can be stated that a response which increases with time in terms of the drop in *F* arises as a consequence of a toxic action which reduces the sperm half-life. But in such a case, the assessment should be based on the variation of the half-life parameter, because the use as response to the *F* drop at a given time necessarily leads to a result in which the effects of the toxic action and the sperm age are confused.

This fact is illustrated in Figure 4(B3), which represents the response to the toxic agent defined as *R*_*F*_—that is, in the appropriate form for model (1)—in the bivariate frame of model (7). In these conditions, if intercept is subtracted to each curve, the fittings to model (1) produce the same *m*_*F*_ values as those obtained using the responses defined with respect to the control at each time, according to the M0 approach. But this perspective makes evident that the fall of the fertilizing capability due to the toxic action begins, at each time, at a different level, determined by the remaining capability of the sperm at this age. Despite the low value of *σ* used, the CI of the parametric estimates obtained with (1) were 10–100 times higher than those produced by (7). The degrees of freedom involved in one and another model justify to a large extent this difference, which to a minor extent is due to the fact that none DR model by itself can explain satisfactorily the behavior of this system. Later, we will see other consequences of the M0 approach.

### 3.2. Effects of the Experimental Error

The same simulations were now performed using five different levels of experimental error [*σ* = 0.050-(0.025)-0.150], under the four conditions resulting from combining single or duplicate observations with raw or smoothed (moving average, window = 3) data.

As expected in the light of the preceding results, model (1) was appreciably more error sensitive. In assays without replicates, the proportion of repetitions with all significant estimates did not reach 100% at any of the times considered, even with the lowest error (*σ* = 0.050). With two replicates, or smoothing without replicates, 100% of significant estimates were reached at the times *t*_3_ and *t*_4_. By combining two replicates with smoothing, also 100% was found at *t*_2_. With *σ* = 0.100, the model was only significant at *t*_2_, *t*_3_, and—with pronounced skewness and kurtosis—*t*_4_, when, besides two replicates and smoothing, the concessive criterion described in the numerical methods section was applied to the CI calculation.

**Figure 5:**
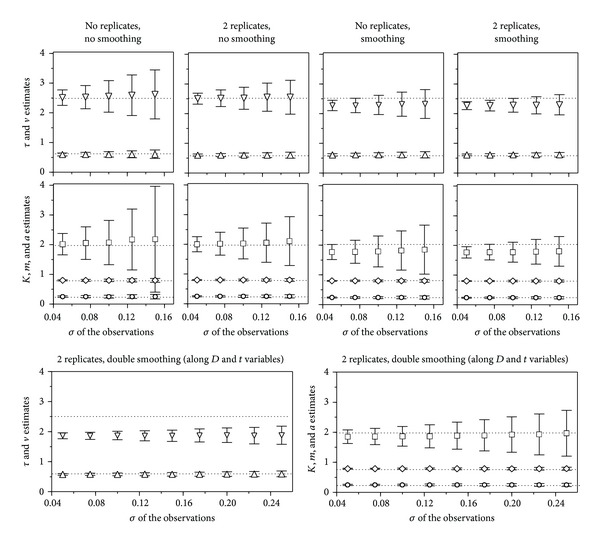
Effect of the observational error on the parametric estimates of model (7) (*τ*: ▵, *v*: ▿, *K*: ⋄, *m*: ∘, *a*: □) and their confidence intervals under the specified conditions. Dotted lines indicate parametric true values.

Model (7) produced, instead, satisfactory fittings in the twenty cases (Figure 5, top). The most error-sensitive parameters were those linked to slopes (*v* and *a*), *τ*, *K*, and *m* being remarkably robust. In the studied range of *σ*, the CI increase of the parametric estimates was only slightly deviated from linearity at the two higher errors in assays without replicates, where the frequency of statistically significant *a* estimates decreased to 99.8 and 99.3%. Otherwise, all the estimates were significant at 100% of the 2,000 repetitions. The use of two replicates reduced markedly the CI (minimal reduction was 27% in *m* and maximal 52% in *a*), increasing at 100% the frequency of the significant estimates of *a* with the highest error. Smoothing along the *D* variable produced equivalent results and, when it was applied with two replicates, the CI reductions varied between 31% (*τ*) and 66% (*a*), all the estimates being significant in 100% of repetitions even if *σ* = 0.200.

**Figure 6:**
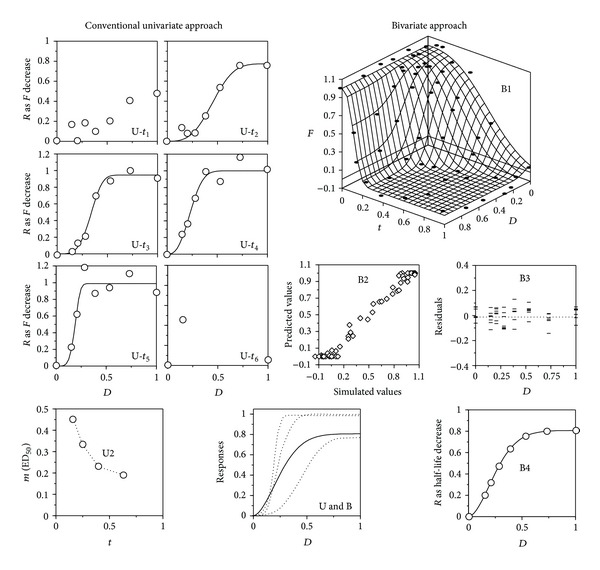
Simulation (points) of a single assay (*σ* = 0.100, 2 replicates, no smoothing) and its fitting (lines) to models (1) (U series) and (7) (B series). In B series, correlation between simulated and predicted results (B2), residuals as a function of the dose (B3) and dr relationships (U and B) according to the univariate (dotted lines) and bivariate (solid line) approaches are also shown. Acceptable fittings were not possible at *t*_1_ and *t*_6_ (omitted points at *t*_6_ were located outside the represented domain) (The rest of keys as in Figure 2). See also text and Table 2.

**Figure 7:**
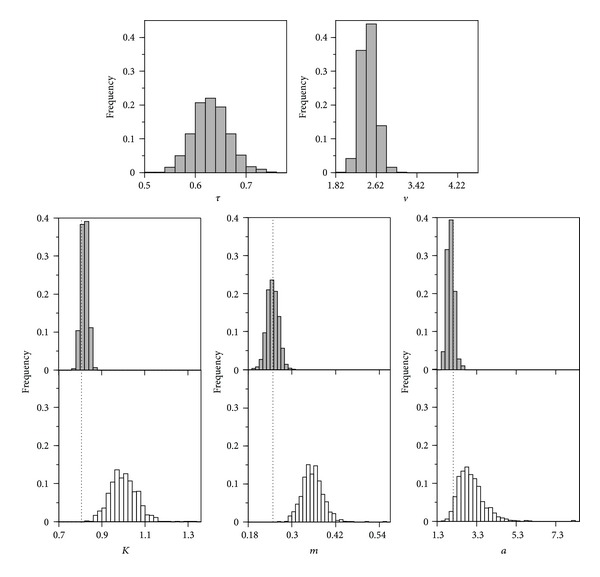
Distributions of the parametric estimates obtained with models (1) (white) and (7) (grey) in 2,000 repetitions of an assay with *σ* = 0.100, 2 replicates and smoothed data along the variable *D*. Dotted lines indicate parametric true values.

Figure 6 and Table 2 illustrate the advantages of the M1 approach supposing a single assay with *σ* = 0.100, two replicates, and no smoothing. Another comparison, using the same error and selecting for M0 the time with the best fit, is that referring to the distributions of the parametric estimates, supposing 2,000 repetitions, two replicates, and smoothing (Figure 7). Even so, M0 produced strongly biased and platykurtic distributions, very problematic in practice.

### 3.3. Smoothing

The moving average method is recommended by some authors [24] to determinate accurately the ED_50_ and, indeed, it reduces the effect of the experimental error, giving statistical significance to estimates which would not have it by using raw data. The drawback is the bias that this method produces in some parametric estimates. In a Weibull function without error, the usual smoothing (window = 3) causes bias only on the parameter *a*, reducing slightly the slope (an effect that is accentuated by higher order windows). In the presence of error, the situation becomes more complex, since smoothing tends to correct the slope increase, statistically associated with the homoscedastic error, but it is easy to realize that it can also lead to overestimate the asymptote, if it is not properly defined by the experimental data (this problem is usually corrected by including the restriction *K* ≤ 1 in the fitting algorithm, although at the risk of biasing the parameters *m* and *a*).

A bivariate model as (7) admits smoothing along either of its two variables, or even both. The above described results were obtained by smoothing along the dose and, as shows the top part of Figure 5, the drawback was an admissible underestimation of the parameters *v* and *a*. Smoothing along the time was less satisfactory for two reasons: the moving average method involved less values and a decreasing sigmoid—first equation of model (7)—proved to be more sensitive than an increasing one to the homoscedastic error (we ignore the cause of this fact, but it was repeatedly confirmed in series of 2,000 runs, with symmetrical curves or not). In any case, this treatment produced a strong bias in the parameter *v* (1.98 instead of 2.50), and weaker in *a*.

When smoothing was applied along both variables (Figure 5, down), the result showed a strong bias in *v* (the least important parameter of the system), but in return it produced, even with *σ* = 0.250, unbiased estimates for the rest of the parameters; all of them (including *v*) are significant in 100% of the repetitions. This means that all toxicological parameters, as well as the sperm half-life, can be estimated with a reasonable accuracy, even when the standard deviation of the observations reaches 25% of their maximum value.

### 3.4. Error and Experimental Effort

It is pertinent to note that the conditions imposed to the described simulations were stricter than the real ones. In practice, the fertilization ratio at time zero is a unique value of the system, independent of the dose and coded as 1. Thus, no initial value can be greater than 1 and, after a certain time, especially in the presence of toxic, values *F*_*c*,*t*_ > 1 are in practice much less probable than those due to a random normal—with mean equal to 0–number generator. Moreover, although a value *F*_*c*,*t*_ > *F*_0*t*_—producing a negative response *R*_*F*_—is possible, a value *F*_*c*,*t*_ < 0 is not. In Figures 6(B1) and 6(B2), it can be seen that a part of the accepted error corresponds to such cases (*F*_*c*,0_ > 1 and *F*_*c*,*t*_ < 0). If simulation includes a condition converting these values into 0 and 1, respectively, the CI produced by model (7), without replicates or smoothing, are reduced 16–20% (the gain is lower in model (1)). This condition, although may be more realistic, was not used.

On the other hand, experimental evidence suggests that the real error in this assay is rather heteroscedastic and stronger in middle than in extreme observational values. Irrespective of using ordinary or weighted least squares as regression method, this condition produces less drastic deviations than those derived from the homoscedastic error applied here.

In any case, to achieve the precision obtained with model (7) without replicates, using model (1)—now ignoring its problematic interpretation—would require at least 4-5 replicates. Thus, a usual assay in the M0 approach, with 8 doses and 4 times, would need at least 128 experimental units. In the M1 approach, 8 × 7 = 56 units would produce at least an equivalent precision and more information, and even the use of two replicates (112 units) would be more economic, with a much higher precision. Moreover, one additional dose improves CI in both approaches, but one additional time only does it in the case of M1, since in M0 it means merely to obtain a new and different DR profile. Finally, the simpler protocol required by the M1 approach minimizes the operative inaccuracies potentially affecting the independent variables.

## 4. Discussion

As a consequence of the drift with time produced by the M0 approach in a toxicological assessment, the *m*_*F*_ values underestimate the toxic potency (regarding *m*_*τ*_ in M1) at short times and overestimate it at longer times. The opposite occurs in the maximum response *K*_*F*_ regarding *K*_*τ*_. The statistically most acceptable fittings are found in general at central times, but even so, the variations in the toxicological parameters are too wide; the best fit is not necessarily the most representative one and there is not a criterion to define* a priori* the most appropriate time.

On the other hand, if the DR curves resulting from the two approaches are compared, it can be stated that M0 tends to underestimate the effects of low doses even when *m*_*F*_ < *m*_*τ*_ at high times (Figure 6(U and B)). Although extremely arguable, two indexes very cited in the ecolegal field [31] are NOEC and LOEC (no observed and lowest observed effect concentration, resp.). Both are obtained by variance analysis, define essentially the limitations of this analysis as a method of toxicological assessment, and are definitely tending to underestimate any toxic effect. This will be underestimated *a fortiori*, if NOEC and LOEC are based on the M0 approach.

**Figure 8:**
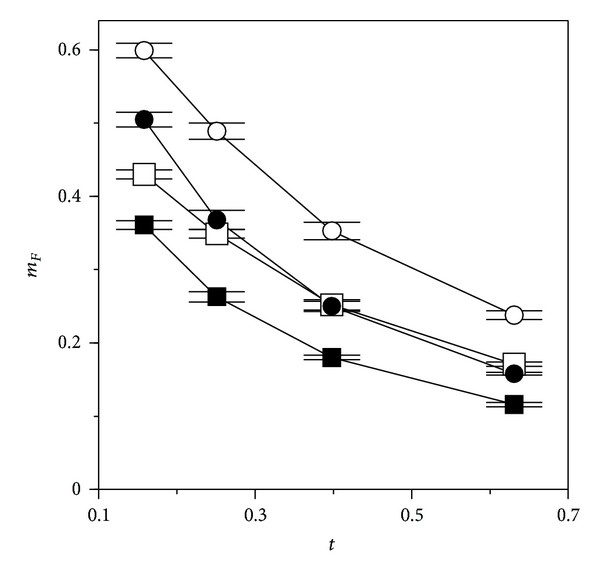
Effect of the spermatic half-life (*τ*) on the estimation of the parameter *m*_*F*_ (ED_50_) by means of model (1) at different times, supposing observations with a negligible error (*σ* = 5 × 10^−4^). Simulations with model (7), combining the following pairs of true parametric values: □: *τ* = 0.6, *m*_*τ*_ = 0.25; ■: *τ* = 0.6, *m*_*τ*_ = 0.35; ◯: *τ* = 0.4, *m*_*τ*_ = 0.25; ●: *τ* = 0.6, *m*_*τ*_ = 0.35 (the rest of the parametric values as in Table 1). Bars indicate the confidence intervals (*α* = 0.05) of the estimates.

Even more controversial is the fact that the results of the M0 approach depend on the sperm half-life *τ* in the assay conditions (in fact, half-life should be included among the standardization needs for M0). Since temperature shortens *τ*, its effect (canceled in M1 by using half-life variations) takes part in M0 through the effect of *τ* on the fertilization ratio, on which the assessment is based (Figure 8). This creates an inevitable and artifactual underestimation of the toxicity at low temperatures, beyond the result of a slower metabolism, and a complementary overestimation at high temperatures. Since bioassays are usually carried out at temperatures close to those characterizing the habitat of the wild animal, the application of M0 will involve a higher legislative tolerance in cold than in warm seas.

## 5. Conclusions

The bioassay studied here is of a special elegance and applicability, which are lost to a great extent because of the use of the fertilization ratio as evaluation criterion. The essence of this issue is the fact that, in a dynamic system, any perturbation cannot be properly characterized through isolate values of any nonlinear-in-time variable but through the variation of some parameter of a model including time in its structure. This conflict, not too rare in physiological contexts, is similar, for example, to that one that arises when a toxic effect on a microbial or cellular batch culture is assessed by using a variable as biomass or some primary metabolite, instead of some parameter of some growth equation.

When this characteristic of the target system is not taken into account, the difficulty to obtain reproducible results often leads to an accumulation of procedural restrictions which only overstandardize the protocol, obstructing its execution without solving the main problem. We believe that sperm bioassays are currently in this condition, despite the existence of very rigorous results about the fertilization kinetics and the factors affecting it, which provide the key to reformulate the toxicological focus in the form proposed here.

Focusing the bioassay on a parameter (the sperm half life) is necessary, first of all, if we want a toxicological assessment with a unique solution and a clear interpretation. It is also more realistic, since the exposure of the gametes to the toxic during only a fraction of their life span has little to do with what occurs in natural conditions. Finally, the procedure is conceptually more direct, experimentally simpler, and more robust against the observational error and variations in the particular variables of the system, and it removes the artifactual effect of temperature.

Using a bivariate model can seem a disadvantage regarding other apparent simpler routines. In this case, however, the current informative means make its application trivial, while the apparently simpler solution requires a more embarrassing protocol and produces more problematic results. DeLean et al. [32] underlined time ago, in a similar context to this, the advisability of “…analyzing all of the curves simultaneously, forcing them to share certain parameters in common.” In agreement with this opinion, we believe [33–36] that bivariate approaches of the type proposed here could improve any bioassay in which the inhibitory or stimulatory action of an effector is superposed on the variation with time of the target system or the particular time course of the response is a relevant aspect of that action. Some bioassays that are based on hemolytic processes could be examples on this matter.

Finally, from the ecotoxicological point of view, it could be pointed out that, in contrast to what seems an implicit assumption, the sperm bioassays are not an alternative to larval ones. In fact, the two types of results can be considered only as two components of the real impact of the toxic under study on the target species. Perhaps this type of assumptions is part of the reasons explaining why the state of many ecosystems is more critical than which is supposed by the parsimony of the respective environment protection policies.

## Figures and Tables

**Table 1:** Properties of the parametric estimates obtained by fitting a virtual assay (2,000 repetitions) to models (1) and (7), the first individually applied at 6 increasing times. Negligible experimental error (homoscedastic σ = 5 × 10^−4^) and no replicates were supposed. CI (%): average confidence interval (α = 0.05) as percentage of the estimate value; STS (%): percentage of statistically significant estimates; SK and KT: skewness and kurtosis coefficients; ALL STS (%): percentage of fittings in which all the estimates were statistically significant; r^2^: correlation coefficient between simulated and predicted values. See also Figure 4.

	True parametric values [4]	Overall fitting to the model [4]	Individual fittings to the model [1]					
			*t*_1_	*t*_2_	*t*_3_	*t*_4_	*t*_5_	*t*_6_
*τ*	0.600	0.600	—	—	—	—	—	—
CI (%)		0.081	—	—	—	—	—	—
STS (%)		100.0	—	—	—	—	—	—
SK		−0.02	—	—	—	—	—	—
KT		0.07	—	—	—	—	—	—

*v*	2.500	2.500	—	—	—	—	—	—
CI (%)		0.105	—	—	—	—	—	—
STS (%)		100.0	—	—	—	—	—	—
SK		0.01	—	—	—	—	—	—
KT		−0.04	—	—	—	—	—	—

*K*	0.800	0.800	0.348	0.742	0.986	1.004	1.002	1.000
CI (%)		0.027	1.60	1.44	1.63	2.05	1.00	0.58
STS (%)		100.0	100.0	100.0	100.0	100.0	100.0	100.0
SK		−0.06	0.07	0.13	−0.07	0.00	−0.05	0.15
KT		0.01	0.16	−0.14	0.05	0.16	0.07	−0.11

*m*	0.250	0.250	0.463	0.430	0.349	0.252	0.171	0.110
CI (%)		0.100	1.33	1.30	1.71	2.61	1.72	5.80
STS (%)		100.0	100.0	100.0	100.0	100.0	100.0	100.0
SK		0.06	0.03	−0.20	0.08	−0.03	−0.06	−0.02
KT		0.07	0.06	0.04	0.11	0.03	−0.05	0.36

*a*	2.000	2.000	4.209	3.942	3.559	3.186	2.790	2.641
CI (%)		0.179	6.48	5.50	7.02	10.12	7.38	17.40
STS (%)		100.0	100.0	100.0	100.0	100.0	100.0	100.0
SK		0.13	0.00	−0.14	0.09	−0.03	−0.19	0.73
KT		0.17	0.07	−0.18	−0.02	0.09	0.12	1.98

All STS (%)		100.0	100.0	100.0	100.0	100.0	100.0	100.0
*r* ^2^		1.000	1.000	1.000	1.000	0.999	1.000	1.000

**Table 2:** Parametric estimates and confidence intervals (as % of the estimate value) obtained by applying uni- and bivariate approaches (1) and (7) to the simulation of a single assay (*σ* = 0.100, two replicates, no smoothing). Results at times *t*_1_ and *t*_6_ did not allow acceptable fittings, and at *t*_5_ the *a* estimate was not significant (CI > 100). See also Figure 8.

True parametric values	Bivariate approach		Uivariate approach at the specified times							
	Estim.	CI (%)	*t*_2_		*t*_3_		*t*_4_		*t*_5_	
			Estim.	CI (%)	Estim.	CI (%)	Estim.	CI (%)	Estim.	CI (%)
*τ* = 0.600	0.603	10.1	—	—	—	—	—	—	—	—
*v* = 2.500	2.635	13.4	—	—	—	—	—	—	—	—
*K* = 0.800	0.807	3.4	0.774	17.3	0.946	8.9	1.000	14.6	0.989	15.3
*m* = 0.250	0.247	13.3	0.451	16.3	0.334	9.0	0.231	20.8	0.190	17.4
*a* = 2.000	1.777	21.5	3.127	53.6	4.097	44.1	2.689	72.3	5.073	>100

*r* ^2^	0.980		0.980		0.989		0.962		0.932	
